# The expanding role of biomarkers in the management of IgA nephropathy

**DOI:** 10.1016/j.kisu.2026.01.004

**Published:** 2026-07-20

**Authors:** Koyal Jain, Dana V. Rizk

**Affiliations:** 1 Division of Nephrology and Hypertension, Department of Medicine, University of North Carolina at Chapel Hill, Chapel Hill, North Carolina, USA; 2 Division of Nephrology, Department of Medicine, Heersink School of Medicine, University of Alabama at Birmingham, Birmingham, Alabama, USA

**Keywords:** biomarkers, diagnosis, IgA nephropathy, noninvasive, prognosis

## Abstract

IgA nephropathy is the most prevalent glomerular disease worldwide. To date, a percutaneous kidney biopsy is needed for diagnosis and assessment of disease activity and prognosis. Histology and generic markers of kidney dysfunction, such as proteinuria, hematuria, and estimated glomerular filtration rate, are commonly used to assess kidney damage. Noninvasive biomarkers that can be used to provide diagnostic and prognostic information, enable risk stratification, guide treatment selection, and help monitor treatment response are greatly needed. On the basis of limited data, several promising novel biomarkers specifically related to IgA nephropathy pathophysiology have been proposed and need to be validated and standardized for use in routine clinical care. Once these biomarkers become available, the hope is that they will lead to early diagnosis of IgA nephropathy and provide more accurate prognosis of disease for individual patients. Given the complexity of the pathogenesis of IgA nephropathy, it seems likely that multiple biomarkers will need to be used to optimize patient care.

The clinical assessment of disease progression and severity in IgA nephropathy (IgAN) has long relied primarily on markers of kidney dysfunction, including estimated glomerular filtration rate (eGFR), proteinuria, and hematuria.^1^, ^2^, ^3^ Although these markers are pivotal in the management and monitoring of disease progression, they do not evaluate the underlying pathophysiological processes specific to IgAN. There is a need to identify markers that reflect the underlying mechanisms of disease in IgAN. The identification and validation of novel biomarkers are key to facilitating screening and diagnosis, better predicting patient prognosis, guiding treatment selection in the face of an increasing number of treatment options, and monitoring treatment efficacy in patients with IgAN.

**Key Learning Points**

• The recent increase in clinical research in IgA nephropathy (IgAN) has provided an ideal opportunity to gather data to help identify, validate, and standardize novel markers that may reflect the underlying mechanisms of disease. It is envisaged that these biomarkers will improve diagnosis and prognosis, enable risk stratification, guide treatment selection, and help monitor treatment response.
• To date, several novel biomarkers specifically relating to IgAN pathophysiology have been proposed on the basis of limited study data; however, none of these are currently available for use in routine clinical practice. Further research is urgently needed.

In recent years, there has been an explosion of clinical research in IgAN, with a large number of clinical trials, both completed and ongoing, investigating a wide range of treatment targets.^4^ This rapid expansion has provided new opportunities to identify and validate novel biomarkers linked to the pathogenesis of IgAN and the underlying mechanisms of the disease. Biomarkers are being identified using novel techniques, such as single-cell transcriptomics, genome-wide association studies, pathomics, proteomics, and metabolomic analyses.^5^, ^6^, ^7^, ^8^, ^9^, ^10^, ^11^

An ideal biomarker for use in clinical practice should be biologically plausible, sensitive and specific, generalizable, minimally invasive to collect, resistant to degradation, and easy and inexpensive to measure.^12^ For the purposes of this article, we define a biomarker as “a biological molecule found in blood, other body fluids, or tissues that is a sign of a normal or abnormal process, or of a condition or disease,” based on the National Institutes of Health National Cancer Institute Dictionary of Cancer Terms.^13^ As such, we exclude eGFR, as it has been established as a direct surrogate marker of kidney function and is, therefore, a definitive end point of IgAN-mediated organ damage. Here, we focus on the markers that precede eGFR decline, including proteinuria, hematuria, and novel biomarkers that relate to the pathogenesis of IgAN.

## Overview of Biomarkers in IgAN

### Histology

Histologic assessments play a key role in IgAN, with a definitive diagnosis being predicated on the presence of dominant or codominant mesangial IgA deposits, determined by immunofluorescent or immunohistochemical staining of kidney biopsy samples.^14^ Additionally, further examination of kidney biopsy samples using the Oxford MEST-C (M = mesangial hypercellularity, E = endocapillary hypercellularity, S = segmental glomerulosclerosis, T = tubular atrophy/interstitial fibrosis, and C = crescents) classification system serves to describe a patient’s specific pathologic features, as recommended in major clinical guidelines.^15^^,^^16^ Components of the MEST-C score have been shown to predict clinical outcomes and eGFR decline, independent of clinical data at the time of biopsy.^15^, ^16^, ^17^ However, although histology is of great prognostic utility at diagnosis, these histologic features are only available through an invasive procedure; as a result, their use is limited by the risks associated with performing biopsies and the willingness of patients to undergo the procedure. Repeat biopsies for the monitoring of disease progression and treatment effect are, therefore, of limited widespread appeal.

The evidence supporting the use of histologic data to guide treatment selection is limited. Of the few repeat biopsy studies that have been conducted in patients with IgAN, some have assessed the impact of immunosuppressive treatment on active glomerular lesions.^18^^,^^19^ One such study suggested endocapillary hypercellularity could be reversed following immunosuppression, whereas another found mesangial proliferation was significantly reduced following immunosuppression and tonsillectomy, with both studies demonstrating improvements in clinical outcomes, such as proteinuria and hematuria.^18^^,^^19^ However, further data from robust randomized clinical trials are needed to fully understand the use of histology in guiding treatment selection in IgAN.^16^^,^^19^

### Generic markers of kidney dysfunction

#### Proteinuria

Proteinuria has long been established as a marker of glomerular damage.^1^ Both cohort studies and randomized clinical trial–level analyses have shown a clear association between proteinuria and kidney outcomes, making it an important prognostic biomarker in IgAN.^20^, ^21^, ^22^ Sustained proteinuria >1 g/d was shown to be a strong independent predictor of the rate of progression of kidney disease and development of kidney failure in individuals with IgAN, and this level of proteinuria (>0.75–1 g/d despite ≥90 days of optimal supportive care) has been used to define people at “high risk” of progressive chronic kidney disease in the Kidney Disease: Improving Global Outcomes (KDIGO) 2021 guidelines.^22^, ^23^, ^24^, ^25^ However, analysis of data from the UK National Registry of Rare Kidney Diseases (RaDaR) of patients with IgAN (2299 adults and 140 children) showed that even patients who were generally perceived as being low risk (proteinuria <1 g/d) in fact experience high rates of kidney failure within 10 years.^26^

Early proteinuria reduction has been established by the US Food and Drug Administration as a “reasonably likely” surrogate end point to assess the effects of a treatment on progression to kidney failure in IgAN and has also been used as a basis for accelerated approval of new IgAN treatments.^21^^,^^27^, ^28^, ^29^ Regarding the assessment of changes in proteinuria over time, a meta-analysis of 13 IgAN trials (N = 1299 patients) reported a positive relationship between treatment effects on 1-year proteinuria reduction and on a composite clinical end point (combination of different end points used in each trial), which was close to reaching statistical significance (*P* = 0.052). However, there was a stronger relationship between treatment effects on 1-year eGFR slope and the composite clinical end point (*P* = 0.001). This result suggests that, although proteinuria reduction remains an important goal and that 1-year proteinuria reduction can be used as a surrogate measure of treatment effectiveness, it should ideally be accompanied by a reduction in the rate of eGFR decline over the same time period.^30^

As mentioned above, treatment decisions for IgAN have typically been driven by the patient’s level of proteinuria, which is also used to define his/her risk of disease progression. However, proteinuria is a nonspecific marker that can result from inflammatory disease activity and/or chronic glomerular damage and, therefore, does not differentiate between active and chronic disease. Additionally, although proteinuria is a noninvasive and inexpensive way to monitor response to treatment, it presents certain challenges, such as variability in time of urine collection (first morning void vs. sporadically timed urine samples) and day-to-day variability, which is affected by diet and exercise habits.^31^ Please see article titled “Management of IgA nephropathy and the expanding role of immunomodulation” by Canetta and Reich^32^ within this supplement for further information on this topic.

#### Hematuria

Hematuria is the most common clinical feature of IgAN and is an early sign of damage to the glomerular capillary basement membrane, crescent formation, or both.^2^^,^^3^^,^^25^^,^^33^, ^34^, ^35^ Although the degree and persistence of hematuria have been shown to be correlated with higher rates of kidney function loss in some studies, the value of hematuria in predicting outcomes in patients with IgAN is debated, partially because of day-to-day variability and a lack of standardized measurements.^25^^,^^33^^,^^34^^,^^36^ Further standardization is required: for example, standardized tests using flow cytometry to detect levels of red blood cells in urine; aligning on what constitutes minimal, mild, moderate, and severe hematuria; and using time-averaged hematuria to account for changes over time in patients with IgAN.^33^^,^^36^

### Biomarkers specifically related to IgAN pathogenesis

The pathophysiology of IgAN has been reviewed earlier in detail in the article titled “IgA nephropathy: an overview of the disease, its pathophysiology, and involvement of the gut-kidney axis” by Cheung and Mariani^37^ within this supplement. We include here a simplified version of disease pathogenesis as it relates to biomarkers.

#### Hit 1: excess production of galactose-deficient IgA1

**Figure 1:**
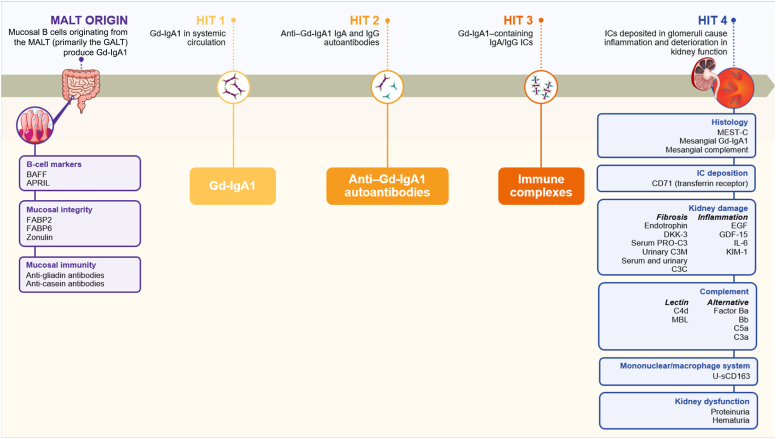
**Overview of IgA nephropathy pathophysiology and potential associated biomarkers.**^9^^,^^38^^,^^39^^,^^42^, ^43^, ^44^, ^45^, ^46^ APRIL, a proliferation-inducing ligand; BAFF, B-cell–activating factor; C, complement component; CD71, cluster of differentiation 71; DKK-3, Dickkopf-3; EGF, epidermal growth factor; FABP, fatty acid–binding protein; GALT, gut-associated lymphoid tissue; GDF-15, growth/differentiation factor-15; Gd-IgA1, galactose-deficient IgA1; IC, immune complex; IL-6, interleukin-6; KIM-1, kidney injury molecule-1; MALT, mucosa-associated lymphoid tissue; MBL, mannose-binding lectin; MEST-C, mesangial hypercellularity, endocapillary hypercellularity, segmental glomerulosclerosis, tubular atrophy/interstitial fibrosis, crescents; U-sCD163, urinary soluble cluster of differentiation 163.

Excess production of galactose-deficient IgA1 (Gd-IgA1), a form of IgA prone to self-aggregation, occurs in the mucosa-associated lymphoid tissue, of which the gut-associated lymphoid tissue is believed to be the largest contributor. Within the gut-associated lymphoid tissue, Gd-IgA1 production is greatest in the Peyer patches of the distal ileum.^38^, ^39^, ^40^, ^41^ Increased levels of circulating Gd-IgA1 are observed in the serum of patients with IgAN and are associated with poor kidney outcomes, including increased proteinuria, disease progression, and kidney failure (Figure 1).^47^, ^48^, ^49^, ^50^, ^51^, ^52^, ^53^ This excess availability of circulating Gd-IgA1 forms “hit 1” of the underlying “4-hit” hypothesis of IgAN pathophysiology.^43^ Serum Gd-IgA1 levels have been shown to differentiate patients with IgAN from healthy controls with a reasonable degree of sensitivity and specificity, suggesting it could be added to a panel of noninvasive diagnostic markers for IgAN.^47^^,^^54^, ^55^, ^56^, ^57^ Furthermore, analysis of sera from patients with IgAN demonstrated higher levels of advanced oxidation protein products compared with healthy controls; when accompanied by elevated Gd-IgA1, this oxidative stress pathway activation was shown to correlate with disease progression.^53^ Evidence from clinical trials, such as Nefecon in Patients with Primary IgA Nephropathy at Risk of Progressing to End-Stage Renal Disease (NefIgArd) (phase 3, Nefecon), ORIGIN (phase 2b, atacicept), RUBY-3 (phase 1b/2a, povetacicept), and ENVISION (phase 2, sibeprenlimab), demonstrates that agents targeting the immunologic elements of IgAN can reduce levels of circulating Gd-IgA1 compared with placebo (Thomas RC, Nawaz N, Barratt J. Specificity of nefecon in targeting pathogenic IgA in IgA nephropathy while preserving systemic humoral immunity [abstract]. Presented at: American Society of Nephrology Kidney Week. October 23–27, 2024; San Diego, CA. Abstract FR-PO894; Madan A, Yalavarthy R, Kim DK, et al. Results from longer follow-up with povetacicept, an enhanced dual BAFF/APRIL antagonist, in IgA nephropathy [RUBY-3 study] [abstract]. Presented at: American Society of Nephrology Kidney Week. October 23–27, 2024; San Diego, CA. Abstract FR-PO854; Khan I, Nawaz N, Jama AAA, et al. Effects of nefecon on hits 1, 2, and 3 of the pathogenic cascade of IgA nephropathy: a full NefIgArd analysis [poster]. Presented at: European Renal Association. June 4–7, 2025; Vienna, Austria. Poster 2642; and Barratt J, Lin C, Nawaz N, et al. Atacicept reduces serum anti-Gd-IgA1 levels in IgAN patients [abstract FC051]. *Nephrol Dial Transplant*. 2022;37:gfac107.003).^58^, ^59^, ^60^, ^61^, ^62^ These same trials also show evidence for proteinuria reduction and eGFR benefit (Thomas RC, Nawaz N, Barratt J. Specificity of nefecon in targeting pathogenic IgA in IgA nephropathy while preserving systemic humoral immunity [abstract]. Presented at: American Society of Nephrology Kidney Week. October 23–27, 2024; San Diego, CA. Abstract FR-PO894; Madan A, Yalavarthy R, Kim DK, et al. Results from longer follow-up with povetacicept, an enhanced dual BAFF/APRIL antagonist, in IgA nephropathy [RUBY-3 study] [abstract]. Presented at: American Society of Nephrology Kidney Week. October 23–27, 2024; San Diego, CA. Abstract FR-PO854; Khan I, Nawaz N, Jama AAA, et al. Effects of nefecon on hits 1, 2, and 3 of the pathogenic cascade of IgA nephropathy: a full NefIgArd analysis [poster]. Presented at: European Renal Association. June 4–7, 2025; Vienna, Austria. Poster 2642; and Barratt J, Lin C, Nawaz N, et al. Atacicept reduces serum anti-Gd-IgA1 levels in IgAN patients [abstract FC051]. *Nephrol Dial Transplant*. 2022;37:gfac107.003).^58^, ^59^, ^60^, ^61^, ^62^ However, there are currently limitations to using Gd-IgA1 as a surrogate biomarker. To begin with, elevated Gd-IgA1 alone is not a cause of IgAN, as first-degree relatives of patients with IgAN can experience increased levels without clinical manifestations of IgAN,^55^ and there is some degree of overlap in Gd-IgA1 levels between patients with IgAN and healthy individuals.^56^^,^^57^ Serum Gd-IgA1 tests alone also do not currently have the sensitivity and specificity needed for real-world clinical use as a diagnostic test in isolation. Furthermore, to date, no extensive mediation analysis has been performed to validate a direct relationship in clinical trials between treatment effect on Gd-IgA1 and kidney function or clinical outcomes.

Given that Gd-IgA1 production has been linked to mucosal surfaces (with the intestinal gut-associated lymphoid tissue being a major source), biomarkers of mucosal integrity, such as intestinal zonulin and fatty acid–binding proteins 2 and 6, and antibodies specific to mucosally encountered antigens, such as gliadin and casein, may also be associated with disease activity and could potentially be used as IgAN-related prognostic biomarkers.^11^^,^^63^, ^64^, ^65^, ^66^

Regulators of B-cell survival, maturation, and homeostasis, such as B-cell–activating factor (BAFF) and a proliferation-inducing ligand (APRIL), play important roles in IgA production.^67^^,^^68^ BAFF levels are associated with increased levels of IgA1 and proteinuria, as well as histologic signs of IgAN and reduced kidney function.^69^, ^70^, ^71^ Similarly, APRIL levels have been associated with increased expression of Gd-IgA1 and worsening kidney disease severity.^69^^,^^72^^,^^73^ With the rise of investigational agents targeting BAFF and/or APRIL for the treatment of IgAN (e.g., atacicept, povetacicept, and sibeprenlimab), several preliminary trials have shown that APRIL inhibition, with or without BAFF inhibition, is associated with reductions in proteinuria and with kidney function benefit (Madan A, Yalavarthy R, Kim DK, et al. Results from longer follow-up with povetacicept, an enhanced dual BAFF/APRIL antagonist, in IgA nephropathy [RUBY-3 study] [abstract]. Presented at: American Society of Nephrology Kidney Week. October 23–27, 2024; San Diego, CA. Abstract FR-PO854).^60^^,^^61^^,^^74^ Given the roles of BAFF and APRIL in IgAN pathogenesis and the preliminary clinical data that are currently emerging, they clearly have potential as noninvasive biomarkers for IgAN and could help inform treatment decisions or monitor treatment response for individual patients in the future.

As mentioned in the article titled “IgA Nephropathy: An Overview of the Disease, Its Pathophysiology, and Involvement of the Gut-Kidney Axis,” microRNAs that regulate gene expression posttranscriptionally are involved in the pathogenic pathway of IgAN.^75^ They can indicate early upstream dysregulation or downstream inflammatory activity.^76^^,^^77^ Circulating microRNAs, such as miR-148b, 374b, and let-7b, directly regulate enzymes involved in O-glycosylation (e.g., core-1-β1,3 galactosyltransferase 1 [C1GALT1]), leading to increased production of Gd-IgA1. Their altered expression in patient serum highlights their potential as early biomarkers of IgAN susceptibility and pathogenesis.^75^^,^^76^^,^^78^

#### Hit 2: anti–Gd-IgA1 autoantibodies

Circulating Gd-IgA1 elicits an autoimmune response by IgG and IgA autoantibodies directed against the poorly O-galactosylated hinge region of the Gd-IgA1 molecule (“hit 2”; Figure 1).^39^^,^^41^^,^^43^^,^^46^ Levels of IgG and IgA specific for Gd-IgA1 are significantly higher in patients with IgAN when compared with both healthy and disease control groups.^54^^,^^79^ However, a proportion of patients with non-IgAN chronic kidney disease had elevated levels of Gd-IgA1–specific IgG (25% of patients) and Gd-IgA1–specific IgA (14%), defined as higher than the 90th percentile for healthy controls. Patients with immune-mediated glomerular diseases other than IgAN in particular also exhibit elevated levels of both these autoantibodies.^54^ As a consequence, the use of Gd-IgA1–specific IgG and IgA autoantibodies as diagnostic biomarkers for IgAN may be limited. Elevated Gd-IgA1–specific IgG provided positive predictive values of 92%, 96%, and 72% and negative predictive values of 89%, 90%, and 82% for IgAN patients compared with healthy controls, patients with nonimmune kidney disease, and patients with immune-mediated kidney disease, respectively. Further assessment of this cohort of patients suggested that the circulating IgG isotype was the predominant autoantibody observed in IgAN following identification of a quantitative association between Gd-IgA1 and Gd-IgA1–specific IgG autoantibodies.^54^^,^^80^ There has been limited research into the differences in IgG subclasses among patients with IgAN. Most recently, a single-center Japanese study of biopsies from 26 patients with IgAN reported that, in those who were IgG positive on immunofluorescence, IgG1 was dominant (74%), followed by IgG2 (37%) and IgG3 (26%).^81^ They also reported a negative correlation between patient age at biopsy and the intensity of IgG1 immunofluorescence, and significantly higher Oxford E and C scores in those with IgG immunofluorescence–positive IgAN than in those without.^81^ Interestingly, no biopsy samples were positive for IgG4. In contrast, however, a Chinese study reported that low levels of IgG4 in the serum were associated with a greater risk of disease progression in patients with IgAN.^82^ Greater understanding of the role of IgG subclasses (and their potential as prognostic markers) in patients with IgAN is, therefore, needed.

Gd-IgA1–specific autoantibodies may have a prognostic role because they have also been found to correlate with lower eGFR and decreased kidney survival in patients with IgAN.^52^^,^^79^^,^^83^ Interestingly, IgG (but not IgA) autoantibody serum levels were correlated with the risk of early recurrence of IgAN in patients who had undergone their first kidney transplantation.^84^ Given these results, further research is required to fully understand the role of these autoantibodies as potential early diagnostic or prognostic biomarkers for IgAN.

Data from the phase 2a JANUS trial have indicated a reduction in Gd-IgA1–specific autoantibodies with atacicept over 72 weeks in patients with IgAN (Barratt J, Lin C, Nawaz N, et al. Atacicept reduces serum anti-Gd-IgA1 levels in IgAN patients [abstract FC051]. *Nephrol Dial Transplant*. 2022;37:gfac107.003). To date, however, the most comprehensive analyses of the effects of IgAN treatment on biomarkers have been in the phase 2 (The Effect of Nefecon in Patients With Primary IgA Nephropathy at Risk of Developing End-stage Renal Disease [NEFIGAN]) and phase 3 (NefIgArd) studies with Nefecon. These 2 randomized clinical trials have also reported a significant reduction in anti–IgA-IgG autoantibodies with Nefecon (16 mg/d); however, no significant change in serum levels of total IgA and IgG up to 12 months after 9 months of treatment with Nefecon was reported (Thomas RC, Nawaz N, Barratt J. Specificity of nefecon in targeting pathogenic IgA in IgA nephropathy while preserving systemic humoral immunity [abstract]. Presented at: American Society of Nephrology Kidney Week. October 23–27, 2024; San Diego, CA. Abstract FR-PO894; and Khan I, Nawaz N, Jama AAA, et al. Effects of nefecon on hits 1, 2, and 3 of the pathogenic cascade of IgA nephropathy: a full NefIgArd analysis [poster]. Presented at: European Renal Association. June 4–7, 2025; Vienna, Austria. Poster 2642).^11^ This reduction in levels of circulating anti–Gd-IgA1 autoantibodies may be a downstream effect of Gd-IgA1 reduction with Nefecon locally within the gut. The lack of significant change in total IgA and IgG suggests that overall systemic humoral immunity was relatively preserved (Thomas RC, Nawaz N, Barratt J. Specificity of nefecon in targeting pathogenic IgA in IgA nephropathy while preserving systemic humoral immunity [abstract]. Presented at: American Society of Nephrology Kidney Week. October 23–27, 2024; San Diego, CA. Abstract FR-PO894).^11^

These findings from both the atacicept and Nefecon trials also suggest that measuring levels of circulating anti-Gd-IgA1-IgG autoantibodies could inform treatment options or monitor response to novel treatments in IgAN, or both.

#### Hit 3: IgA1-containing immune complexes

Gd-IgA1 and anti–Gd-IgA1 autoantibodies form circulating immune complexes (“hit 3”; Figure 1).^39^^,^^41^^,^^43^ However, the presence of these circulating complexes does not only occur in patients with IgAN, having been detected in people without kidney disease.^49^ To date, there have been few assessments of whether these immune complexes could act as biomarkers in IgAN. Only 1 small study, consisting of 50 patients in Japan, demonstrated that serum levels of Gd-IgA1–containing immune complexes were significantly associated with the degree of proteinuria and hematuria in patients with IgAN.^85^

Recent trials assessing agents for the immunologic treatment of IgAN, including Nefecon and sibeprenlimab, have both shown reduction of circulating immune complexes in patients with IgAN who were assigned to the active treatment arms (Thomas RC, Nawaz N, Barratt J. Specificity of nefecon in targeting pathogenic IgA in IgA nephropathy while preserving systemic humoral immunity [abstract]. Presented at: American Society of Nephrology Kidney Week. October 23–27, 2024; San Diego, CA. Abstract FR-PO894; and Barratt J, Mathur M, Liew A, et al. The anti-APRIL antibody sibeprenlimab reduces circulating immune complexes in patients with IgAN: the phase 2 ENVISION randomized controlled trial [abstract]. Presented at: American Society of Nephrology Kidney Week. October 23–27, 2024; San Diego, CA. Abstract FR-OR59).^11^ Further research into whether these IgA1-containing immune complexes can be used as a biomarker in IgAN is required.

#### Hit 4: deposition of immune complexes in the glomerular mesangium

Once immune complexes reach the kidney, they tend to deposit within the glomerular mesangium (“hit 4”; Figure 1).^43^ In turn, this deposition leads to mesangial cell and complement activation, leading to kidney damage downstream. Indeed, a greater intensity of mesangial deposits of Gd-IgA1 has been shown to correlate with lower eGFR in patients with IgAN.^52^

The mesangial cell activation triggered by immune complex deposition possibly occurs through receptors like the transferrin receptor (cluster of differentiation 71 [CD71]).^43^^,^^86^ This binding enhances CD71 expression, which creates a positive feedback loop that results in the overexpression of CD71 in patients with IgAN.^43^^,^^87^ A study of 282 patients with biopsy-proven IgAN reported significantly higher CD71 mRNA expression levels in patients with disease progression (≥30% eGFR decline from baseline) versus nonprogressors; mRNA levels were also significantly higher in nonprogressing patients with IgAN versus controls with no histologic abnormalities.^88^ Immunohistochemistry identified more intense glomerular CD71 protein staining in progressors versus nonprogressors,^88^ and a multivariable Cox model also identified higher CD71 expression levels as a predictor of disease progression.^88^ These findings (and other findings within the study) suggest that increased CD71 expression may be a potential prognostic marker in patients with IgAN.

The complement activation that is triggered by immune complex deposition (Figure 1) results in the release of proinflammatory and profibrotic mediators that lead to glomerular and tubulointerstitial injury.^40^^,^^41^ In patients with IgAN, markers of alternative (factor Ba, Bb, C5a, and C3a) and lectin (C4d and mannose-binding lectin) complement pathway activation are associated with elevated Gd-IgA1 levels.^89^, ^90^, ^91^, ^92^ Histologic evidence has shown deposition of complement proteins in the glomeruli of patients with IgAN, with the intensity of mesangial complement protein deposition being associated with kidney function deterioration.^93^, ^94^, ^95^, ^96^, ^97^, ^98^ Few complement assays are clinically available, and more comprehensive assessments of serum and urine complement components have not been systematically undertaken in patients with IgAN. Further research will be required to validate these potential prognostic biomarkers in IgAN and hopefully identify promising novel molecules that can be easily measured in biofluids.

Monocyte/macrophage infiltration is also thought to play an important role in the progression of IgAN (although the exact mechanism is unclear), with a positive correlation noted between the number of macrophages present per glomerulus and the degree of hematuria observed.^99^ M2 (CD163^+^) macrophages are found in the kidneys of patients with IgAN and are associated with the presence of crescents and lower eGFR.^100^^,^^101^ These macrophages also shed CD163 into the urine, and urinary soluble CD163 has recently been shown to predict the response to systemic glucocorticoid treatment in patients with IgAN.^102^^,^^103^ In the Therapeutic Effects of Steroids in IgA Nephropathy Global (TESTING) trial, patients were found to have a significant decline in urinary soluble CD163 levels at 6 and 12 months in response to methylprednisolone when compared with placebo (79% vs. 37% [*P* < 0.001], and 65% vs. 34% [*P* < 0.001], respectively), and reduced urinary soluble CD163 levels resulted in a lower risk of kidney failure.^103^ This suggests that urinary soluble CD163 may be a promising prognostic biomarker in addition to predicting response to systemic glucocorticoids.

Markers of kidney inflammation and tubular damage that occur following mesangial IgA deposition, such as urinary epidermal growth factor, growth/differentiation factor-15, interleukin-6, and kidney injury molecule-1, have also been shown to be predictive of proteinuria, kidney function decline, and adverse kidney outcomes in IgAN.^104^, ^105^, ^106^, ^107^ Biomarkers associated with kidney fibrosis, including serum and urinary endotrophin and Dickkopf-3, and collagen type III propeptides (serum PRO-C3, serum and urinary C3C, and urinary C3M) have also been identified in IgAN.^108^, ^109^, ^110^

Renal and urinary microRNAs, including miR-21, miR-29, and members of the miR-200 family, are consistently upregulated in association with mesangial proliferation, interstitial fibrosis, and progressive renal damage. These microRNAs may serve as prognostic indicators of disease activity and long-term outcomes in IgAN.^77^^,^^111^, ^112^, ^113^ All of these will require further validation.

## Implications of IgAN Biomarkers for Clinical Practice

### Early diagnosis

Identifying biomarkers for IgAN may allow screening of individuals to detect kidney disease earlier, particularly in populations with a high prevalence of IgAN. Countries such as Japan, Singapore, South Korea, and Taiwan have seen increased rates of diagnosis in part because of universal urine testing programs identifying microhematuria and proteinuria early.^114^, ^115^, ^116^ However, as noted previously, IgAN diagnosis must currently be confirmed by kidney biopsy.

Disease-specific genetic risk scores may further help accurately diagnose IgAN early. For example, a UK-based study calculated an IgAN genetic risk score from single-nucleotide polymorphisms known to be associated with IgAN, and used it to estimate that 19% of White Europeans with unspecified hematuria in their sample had suspected undiagnosed IgAN.^117^

### Prognosis

The International IgA Nephropathy Prediction Tool provides a method to estimate the individualized risk of a 50% decline in GFR, or end-stage kidney disease up to 7 years from diagnosis, and can be used to reevaluate patients at 1 or 2 years after biopsy. This tool currently uses demographic characteristics, histologic data, eGFR, and the urine protein-to-creatinine ratio.^118^^,^^119^ However, this tool has not been validated for making treatment decisions in IgAN.^17^ The discovery and validation of the prognostic capabilities of additional biomarkers could be incorporated into future predictive algorithms to strengthen their accuracy or allow them to be used at time points beyond diagnosis. For example, 4 microRNAs (miR150-5p, miR155-5p, miR 146b-5p, and miR135a-5p) have been reported to increase the discrimination score of the IgAN Prediction Tool, with their expression correlating significantly with the T-lesion of the Oxford classification, as well as with proteinuria and eGFR.^120^ Therefore, an ability to predict outcomes even more accurately and over a longer time horizon with these tools would be desirable and would appear to be within reach.

### Informing treatment selection and measuring treatment effect

There is significant heterogeneity in the clinical presentation and histologic characteristics of patients with IgAN.^121^^,^^122^ Although data are currently lacking, biomarkers could be used to further stratify patients with IgAN based on their biological characteristics. They could also be used to predict the likelihood of clinical remission.

Further evidence from clinical trials, along with novel biomarker data, may provide an opportunity to assess whether a therapy has a greater treatment effect in a given patient subpopulation. This would be beneficial to guide treatment selection and personalization, allowing for treatment effect monitoring and informing individualized treatment decisions, such as optimal dosing, duration, and the potential need for more aggressive combination therapy. A greater understanding of the changes in biomarkers during the disease course of IgAN over a patient’s lifetime could also help identify the most appropriate point to start, pause, stop, or amend the dosing, or even to switch treatments if a patient is not responding. Interestingly, clinical trials are assessing biomarkers in IgAN based on key pathogenic mediators, such as mucosal immunity, kidney inflammation, and fibrosis, which aim to further explore the mechanism by which treatments exert their effects and whether these biomarkers can be used in treatment selection and monitoring of response (Thomas RC, Nawaz N, Barratt J. Specificity of nefecon in targeting pathogenic IgA in IgA nephropathy while preserving systemic humoral immunity [abstract]. Presented at: American Society of Nephrology Kidney Week. October 23–27, 2024; San Diego, CA. Abstract FR-PO894).^11^

### Validation of biomarkers

Although currently limited, the recent increase in clinical trials in patients with IgAN has the potential to provide greater amounts of data on the association between treatment effects on biomarkers and clinical outcomes or surrogate markers, such as eGFR. With sufficient data, meta-analyses could be conducted in the future to help validate these associations. Mediation analyses, in particular, would help determine whether direct effects of novel biomarkers on clinical outcomes can be identified. The validation and standardization of novel biomarkers as predictive of clinical outcomes or reduction in eGFR could be incorporated into regulatory considerations to accelerate drug approvals, as has been done for proteinuria.^21^^,^^25^

### Opportunities to identify future innovative biomarkers

Further research investigating the molecular mechanisms underpinning IgAN may, in the future, lead to the identification of novel biomarkers. Omics studies, such as genomics, transcriptomics, epigenomics, pathomics, proteomics, and metabolomics, can help identify molecular biomarkers that could offer precise stratification of patients, increase understanding of pathogenic mechanisms of the disease, and lead to a more thorough understanding of the molecular characteristics of kidney damage, all of which can support personalized management of patients with IgAN.^5^, ^6^, ^7^, ^8^, ^9^, ^10^, ^11^, ^12^

## Conclusions

Although validated markers of kidney disease progression exist, there is an additional need to identify, fully validate, and standardize biomarkers that are specifically related to IgAN pathogenesis. The recent explosion in clinical research in IgAN provides an opportunity to gather sufficient data to identify and validate novel markers so that they are widely available for clinical use. IgAN has a complex pathogenesis; therefore, it is likely that multiple biomarkers will be needed to better understand a patient’s diagnosis, prognosis, and risk stratification to monitor the course of the disease, treatment selection, and treatment response.

## Disclosure

This article is published as part of a supplement sponsored by Calliditas Therapeutics, an Asahi Kasei company.

KJ has received travel support from the International Society of Glomerular Diseases; and serves as principal site investigator for the FSGSALLAGE (NCT04065438) and FSGS Pediatric (NCT02235857) clinical trials. She also serves as a consultant for Amgen. DVR has grants (pending/received) and research support from Calliditas Therapeutics (Pharmalink), Chinook Pharmaceuticals, LaRoche, Otsuka Pharmaceuticals (Visterra), Pfizer Pharmaceuticals, Reata Pharmaceuticals, Travere Therapeutics (Retrophin), Vera Therapeutics, and Vertex Pharmaceuticals; consulting fees from BioCryst, Biogen, Calliditas Therapeutics (Pharmalink), Chinook Pharmaceuticals, Chugai, Eledon Pharmaceuticals, George Clinical, LaRoche, Novartis, Otsuka Pharmaceuticals (Visterra), Timberlyne Therapeutics, and Vera Therapeutics; honoraria from Alpine Immune Science, Argenx, BioCryst, Calliditas Therapeutics (Pharmalink), Chinook Pharmaceuticals, GlaxoSmithKline, Otsuka Pharmaceuticals, and Vera Therapeutics; and travel support from Calliditas Therapeutics, Novartis, and Otsuka Pharmaceuticals. She also has ownership in Reliant Glycosciences LLC.
